# Identification of highly conserved, serotype-specific dengue virus sequences: implications for vaccine design

**DOI:** 10.1186/s12864-019-6311-z

**Published:** 2019-12-24

**Authors:** Li Chuin Chong, Asif M. Khan

**Affiliations:** Centre for Bioinformatics, School of Data Sciences, Perdana University, Jalan MAEPS Perdana, 43400 Serdang, Selangor Darul Ehsan Malaysia

**Keywords:** Dengue virus, Cross-reactivity, Serotype-specific, Sequence conservation, Entropy, Mutual information, Immune targets, Vaccine design

## Abstract

**Background:**

The sequence diversity of dengue virus (DENV) is one of the challenges in developing an effective vaccine against the virus. Highly conserved, serotype-specific (HCSS), immune-relevant DENV sequences are attractive candidates for vaccine design, and represent an alternative to the approach of selecting pan-DENV conserved sequences. The former aims to limit the number of possible cross-reactive epitope variants in the population, while the latter aims to limit the cross-reactivity between the serotypes to favour a serotype-specific response. Herein, we performed a large-scale systematic study to map and characterise HCSS sequences in the DENV proteome.

**Methods:**

All reported DENV protein sequence data for each serotype was retrieved from the NCBI Entrez Protein (nr) Database (txid: 12637). The downloaded sequences were then separated according to the individual serotype proteins by use of BLASTp search, and subsequently removed for duplicates and co-aligned across the serotypes. Shannon’s entropy and mutual information (MI) analyses, by use of AVANA, were performed to measure the diversity within and between the serotype proteins to identify HCSS nonamers. The sequences were evaluated for the presence of promiscuous T-cell epitopes by use of NetCTLpan 1.1 and NetMHCIIpan 3.2 server for human leukocyte antigen (HLA) class I and class II supertypes, respectively. The predicted epitopes were matched to reported epitopes in the Immune Epitope Database.

**Results:**

A total of 2321 nonamers met the HCSS selection criteria of entropy < 0.25 and MI > 0.8. Concatenating these resulted in a total of 337 HCSS sequences. DENV4 had the most number of HCSS nonamers; NS5, NS3 and E proteins had among the highest, with none in the C and only one in prM. The HCSS sequences were immune-relevant; 87 HCSS sequences were both reported T-cell epitopes/ligands in human and predicted epitopes, supporting the accuracy of the predictions. A number of the HCSS clustered as immunological hotspots and exhibited putative promiscuity beyond a single HLA supertype. The HCSS sequences represented, on average, ~ 40% of the proteome length for each serotype; more than double of pan-DENV sequences (conserved across the four serotypes), and thus offer a larger choice of sequences for vaccine target selection. HCSS sequences of a given serotype showed significant amino acid difference to all the variants of the other serotypes, supporting the notion of serotype-specificity.

**Conclusion:**

This work provides a catalogue of HCSS sequences in the DENV proteome, as candidates for vaccine target selection. The methodology described herein provides a framework for similar application to other pathogens.

## Background

Dengue virus (DENV), a member of the family *Flaviviridae* [1]*,* is a significant infliction that affects approximately 400 million people worldwide, annually [2–4]. The virus is primarily transmitted by mosquitoes of the genus *Aedes*. The arthropod-borne viral infection mostly occurs in tropical and sub-tropical regions, with rural communities increasingly being affected [2, 5]. Notably, over half a million hospitalised cases and approximately 12,500 deaths are reported each year [3]. DENV-associated deaths are closely linked to the severe dengue hemorrhagic fever (DHF) or often the fatal dengue shock syndrome (DSS).

DENV genome is a ~ 11 kb positive stranded RNA, which encodes for a polypeptide that comprises of ~ 3400 amino acids [6]. The polypeptide is cleaved into three structural (capsid protein, C; precursor membrane/membrane protein, prM/M; and envelope protein, E) and seven non-structural (NS1, 2a, 2b, 3, 4a, 4b, and 5) proteins. DENV, being an RNA virus, exhibits a high mutation rate due to the lack of 3′ to 5′ exonuclease proofreading mechanism [7, 8]. There are four distinct, yet closely related serotypes of the virus (DENV1–4) in circulation [2, 9, 10]. A fifth serotype has been reported [11], which follows a sylvatic cycle and is not endemic in human populations, unlike the other four serotypes, and thus, is not considered for analysis herein. The four established serotypes (DENV1–4) share a high degree (~ 65–70%) of sequence similarity between the genomes [12, 13], with average sequence identity between the proteomes of ~ 39–79% [14]. The accumulation of mutation and recombination can facilitate the generation of novel mutants, resulting in the existence of a mutant spectra that collectively can create a quasispecies population within an individual [8, 15–17]. A primary infection by a given dengue serotype generally provides future protective immunity against the particular serotype for the patient. However, this may not be the case with heterologous serotypes during a secondary infection where the memory response is exposed to altered peptide ligands (APLs), a phenomenon often referred to as “original antigenic sin” and is highly associated with DENV2 and 4 [18].

The adaptive immune system, both cellular and humoral, has an essential protective role in DENV infection. A plethora of studies have indicated that DENV CD8^+^ and CD4^+^ T cells play a significant role in controlling DENV infection, either, respectively, through lytic activity against DENV-infected cells and secreting interferon (IFN)-γ or recruiting B-cells and promoting the memory response [19–24]. The cellular response is directed against short peptides derived from proteolysis of self and foreign proteins. These peptides are presented by the major histocompatibility complex (MHC) molecules, referred to as human leukocyte antigen (HLA) molecules in humans, for recognition by the T-cell receptor (TCR) in the form of a ternary complex. Peptides that elicit an immune response are referred to as T-cell epitopes. HLA binding by a peptide is a pre-requisite for determining a T-cell epitope, however binding alone is not sufficient because epitope immunogenicity is also contingent on antigen processing and recognition by a cognate TCR [25]. Sequence diversity among viral proteins, in particular of RNA viruses, can facilitate escape from immune recognition, and thus is a challenge for the development of a tetravalent vaccine. The viral diversity can give rise to one or more amino acid differences where the peptides harboring them can function as alternative T-cell epitopes to the original epitope, and affect the anti-dengue host response. The substitutions, even of a single amino acid, create altered peptide ligands (APLs) that can impair the function of the T-cell through a variety of ways [26–30]. This may include T-cell epitopes that result in a serotype-specific or cross-reactive response, with the possibility of a deleterious outcome that may play a role in DHF/DSS [31–34]. The consideration of APLs may have an important implication and consequence to the safety and efficacy of vaccines in trial.

Khan et al. [35] performed a large-scale identification and analysis of evolutionarily highly conserved amino acid sequences for the entire DENV proteome. They identified 44 pan-DENV sequences, of length 9 to 22 amino acids each that were common across the four serotypes and highly conserved within each, and most were immune-relevant. The pan-DENV sequences may be of utility in the design of tetravalent vaccine to avoid regions of T-cell immunity that are highly variable across the four serotypes, except when they are serotype-specific [33, 36]. In this study, we aimed to identify highly conserved, serotype-specific (HCSS) DENV peptides that are potentially immune-relevant. This is in contrast to the approach by Khan et al. [35], cataloguing pan-DENV sequences as potential vaccine targets. Alternatively, HCSS sequences may also be attractive candidates for vaccine design as such sequences minimise the issue of altered peptide ligands (APLs) that are cross-reactive between the dengue serotypes.

## Method

### Methodology overview

**Fig. 1:**
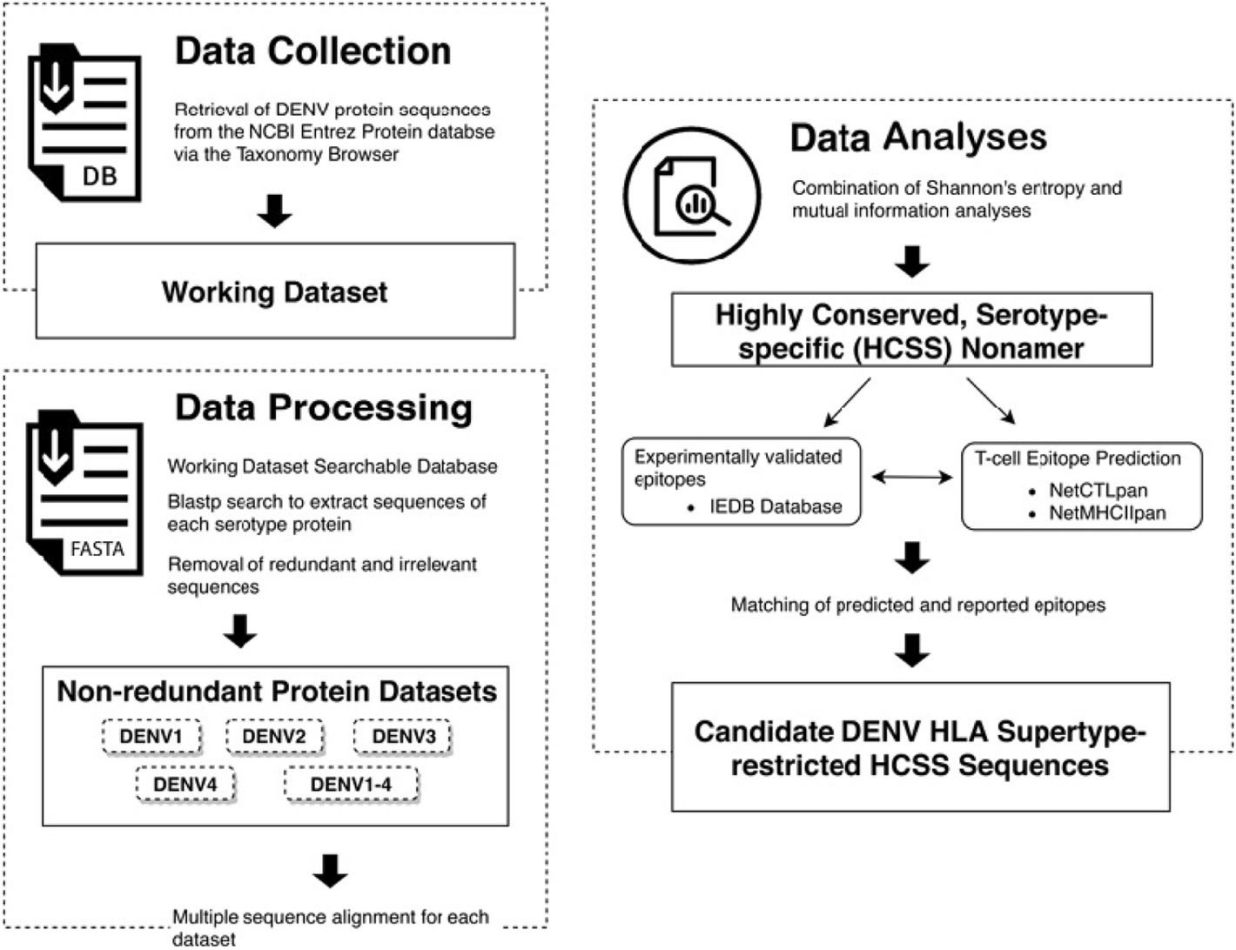
Overview of the methodology employed for the identification and analyses of highly conserved, serotype-specific (HCSS) DENV sequences

The methodology adopted in this study is summarised in Fig. 1. It comprises of three components, namely i) data collection, ii) data processing, and iii) data analyses: identification and characterisation of HCSS sequences.

### Data collection and processing

All DENV protein sequence records were retrieved from the National Center for Biotechnology Information (NCBI) Entrez Protein (nr) database for all dengue serotypes, via the NCBI Taxonomy Browser using the taxonomy identifier (ID) “12637”. Given the polyprotein nature of the DENV translated genome, the database records can contain the protein sequence labelled as a “genome polyprotein” (containing all the 10 proteins), “partial polyprotein” (with at least two to as many as nine proteins, either full-length or partial for the termini proteins) or as a single mature protein [37]. In contrast, influenza A virus sequence records contain data for a single protein given the segmented nature of the genome. The basic local alignment search tool (BLAST; [38]) was used to create a searchable database using the collected sequences. BLASTp search [39] was performed against the local database using a reference sequence for each serotype protein retrieved from the highly curated UniProt database [40, 41]: DENV1, P33478; DENV2, P07564; DENV3, P27915; and DENV4, P09866. The blast parameters (E-value less than 0.05) were used to evaluate the significance of the hits and select the sequences for each serotype protein.

Duplicate sequences, either full-length or as partial sub-sets to the other sequences, were removed to minimise sampling bias. Each serotype protein sequences were then multiple sequence aligned by use of the “Multiple Alignment using Fast Fourier Transform” (MAFFT) tool [42]. Additionally, the non-redundant sequences of the same protein from each of the serotypes were copied into a separate file as a combined dataset of the same protein from the different serotypes, which was also aligned. All sequence alignments were manually inspected for misalignments and were corrected where necessary [43, 44].

### Identification of highly conserved, serotype-specific (HCSS) sequences

Shannon’s entropy and mutual information (MI) analysis were performed using the Antigenic Variability Analyser tool (AVANA) to measure the diversity of DENV proteome within the serotypes (intra-type) and across the serotypes (inter-type), respectively [45–47]. Shannon’s entropy was measured for overlapping nonamer (*1–9, 2–10,* etc.) windows of the aligned sequences. Nonamer length was chosen as it represents the typical length of HLA class I epitopes and the core of class II epitopes. Applying Shannon’s formula, the nonamer peptide entropy *H(x)* at any given position *x* in the alignment was computed by:

$$ H(x)=-\sum \limits_{i=1}^{n(x)}{p}_{i,x}{\log}_2\left({p}_{i,x}\right) $$

where *p(i, x)* is the probability of a particular nonamer peptide *i,* with a starting position *x*. Positions with a high conservation will yield low entropy value and the lowest value, zero, is observed at completely conserved positions. In contrast, a high entropy value indicates a highly variable position, up to a maximum of ~ 39. Only sequences that contained a valid amino acid at position *x* were used for the entropy computation. Positions where more than 50% of sequences contained a gap were discarded. Sequence count in the alignment affects the entropy calculation due to the inverse relationship between sample size and alignment bias [48]. This allows a correction for size bias by applying to each alignment a statistical adjustment, using linear regression that estimates entropy values for an infinite-size sets of sequence [35].

MI analysis is a measure of the dependence between two variables (A and B), which is defined by:

$$ \mathrm{MI}\left(\mathrm{A},\mathrm{B}\right)=\mathrm{H}\left(\mathrm{A}\right)+\mathrm{H}\left(\mathrm{B}\right)-\mathrm{H}\left(\mathrm{A},\mathrm{B}\right) $$

where the joint entropy between two variables is shown as H(A,B). The value is computed by use of the entropy formula by substituting *i* with (A,B), which is the set of all unique pair of values. The high difference between the two datasets yields a high MI value (maximum of 1), while low MI value, approaching zero, exhibits similar distributions of amino acid in the two sets.

A combination of entropy and mutual information analyses were used to identify the HCSS DENV sequences by use of AVANA. The tool requires a metadata with annotated fields for subset selection in a master alignment (a tab delimited alignment file). The combined dataset of the same protein from the different serotypes was used for this purpose as the master alignment, given that protein sequences from the four serotypes were co-aligned to facilitate the comparative analysis. The window size was set to nine amino acids for immunological applications. When a particular serotype was being characterised, the remaining three of the serotypes were combined and selected as the reference set for alignment comparison with the given serotype; all this was done using the metadata subset-selection feature of AVANA. For instance, when DENV1 subset was chosen as the characterised set, the combination of DENV2, 3 and 4 subsets served as the reference set. Nonamers were identified and catalogued as HCSS if they matched the selection criteria of entropy less than 0.25 and MI greater than 0.8. HCSS nonamers that overlapped by at least one amino acid were concatenated to form longer sequences.

### Functional analysis of the HCSS sequences

The functional domains and motifs within each of the HCSS sequences were searched by use of protein function prediction tools, Conserved Domain Database (CDD) [49], Pfam [50] and ScanProsite [51].

### Identification of predicted and known T-cell epitopes

Promiscuous T-cell epitopes restricted to human leukocyte antigen (HLA) class I and class II supertypes were predicted by use of NetCTLpan 1.1 and NetMHCIIpan 3.2 servers, respectively [52, 53]. Supertypes are groups of HLA molecules that share similar peptide binding specificity despite different binding repertoires [54, 55], and thus promiscuous epitopes are the best candidate epitopes for broad population coverage. These two prediction tools have been benchmarked to be among the best performing prediction servers publicly available [56, 57].

With the importance of C-terminal proteasomal cleavage, transporter associated with antigen processing (TAP) transport, and the HLA class I binding in the recognition of cytotoxic T lymphocytes (CTL; T cells’ subgroup), NetCTLpan 1.1 integrates all predictions in the identification of predicted CTL immunogenic epitopes. Predictions were carried out for eight HLA class I representative supertypes of HLA-A and HLA-B genes (HLA-A: A1, A2, A3; HLA-B: B7, B27, B44, B58, B62) with the default settings used [58, 59]. Since the tools did not predict for supertypes directly, this was evaluated manually. Prediction was made for all the representative alleles of each supertype as defined by Sidney et al. [59], and a nonamer was considered to be supertype-restricted if it was predicted positive for at least half of the alleles.

The representative alleles of the supertypes are: **A1:** HLA-A*0101, HLA-A*2601, HLA-A*2602, HLA-A*2603, HLA-A*3002, HLA-A*3003, HLA-A*3004 and HLA-A*3201; **A2:** HLA-A*0201, HLA-A*0202, HLA-A*0203, HLA-A*0204, HLA-A*0205, HLA-A*0206, HLA-A*0207, HLA-A*0214, HLA-A*0217, HLA-A*6802 and HLA-A*6901; **A3:** HLA-A*0301, HLA-A*1101, HLA-A*3101, HLA-A*3301, HLA-A*3303, HLA-A*6601, HLA-A*6801 and HLA-A*7401; **B7:** HLA-B*0702, HLA-B*0703, HLA-B*0705, HLA-B*1508, HLA-B*3501, HLA-B*3503, HLA-B*4201, HLA-B*5101, HLA-B*5102, HLA-B*5103, HLA-B*5301, HLA-B*5401, HLA-B*5501, HLA-B*5502, HLA-B*5601, HLA-B*6701 and HLA-B*7801; **B27:** HLA-B*1402, HLA-B*1503, HLA-B*1509, HLA-B*1510, HLA-B*1518, HLA-B*2702, HLA-B*2703, HLA-B*2704, HLA-B*2705, HLA-B*2706, HLA-B*2707, HLA-B*2709, HLA-B*3801, HLA-B*3901, HLA-B*3902, HLA-B*3909, HLA-B*4801 and HLA-B*7301; **B44:** HLA-B*1801, HLA-B*3701, HLA-B*4001, HLA-B*4002, HLA-B*4006, HLA-B*4402, HLA-B*4403 and HLA-B*4501; **B58:** HLA-B*1516, HLA-B*1517, HLA-B*5701, HLA-B*5801 and HLA-B*5802 and **B62:** HLA-B*1501, HLA-B*1502, HLA-B*1512, HLA-B*1513, HLA-B*4601 and HLA-B*5201.

HLA class II T-cell epitopes were only evaluated for HLA-DR gene, given the ~ 94.7% population coverage [60]. The prediction was done for peptides of length nine and for the three common sub-classes of HLA-DR supertype (Main DR, DR4, DRB3) [61]. The allele restrictions for each of the sub-classes are: **Main DR:** HLA-DRB1*0101, HLA-DRB1*0701, HLA-DRB1*0901, HLA-DRB1*1101, HLA-DRB1*1201, HLA-DRB1*1501 and HLA-DRB5*0101; **DR4:** HLA-DRB1*0401, HLA-DRB1*0405, and HLA-DRB1*0802; **DRB3**: HLA-DRB1*0301, HLA-DRB1*1302, HLA-DRB3*0101, HLA-DRB3*0202 and HLA-DRB4*0101. Class II epitopes are longer (13-25aa) [62] than class I epitopes, and thus a caveat is that the prediction of binders for length nine may not completely capture the essence of CD4^+^ epitope.

Experimentally determined T-cell epitopes of dengue virus were searched for and retrieved from the Immune Epitope Database and Analysis Resource (IEDB) (as of April 2019) [63]. Only the linear T-cell epitopes from positive assays and MHC ligand assays were downloaded and compared with the predicted epitopes.

Separately, a structure-based docking approach was performed to further assess the predictive reliability of the sequence-based approach. A Fast Fourier Transform (FFT) based rigid docking approach by use of ClusPro [64–66] was carried out for a representative HCSS nonamer with a structure template available in PDB (PDB ID: 2JLQ) [67], modelled using SWISS-MODEL [68] against a HLA-A2*0201 structure, also available in PDB (PDB ID: 2GIT) [69]. A known peptide-HLA complex (PDB ID: 3SPV) was used as a positive control.

## Result

### Data collection and processing

The NCBI Entrez Protein Database (nr) comprised of a total of 19,432 DENV protein sequence records (as of April 2018): DENV1 (6,531), DENV2 (6,404), DENV3 (4,301), and DENV4 (2,196). The discrepancy in numbers is a reflection of dengue serotype distribution in nature and sequencing efforts to study the virus [70]. A total of 63,890 individual protein sequences were extracted from the records given the polyprotein nature of many of the sequences in the records. Compared to the number of redundant sequences (12,404) collected by Khan et al. [35] (as of 2007), the increase was significant more than a decade later, up to ~ 415% (by 51,486 sequences; average of ~ 37% per year) (Table 1). However, after the removal of duplicate sequences, only a total of 13,648 non-redundant sequences remained, which is a striking drop of ~ 78.64%: DENV1 (4,297), DENV2 (5,020), DENV3 (2,978) and DENV4 (1,353). The protein NS5 had the least fraction of redundant sequences (~ 63%) across the four serotypes, while NS2b and NS4a had the most (~ 92%).

**Table 1:** Number and distribution of redundant (R) and non-redundant (NR) reported DENV protein sequences in 2007 and 2018

Protein / Serotype	DENV1		DENV2		DENV3		DENV4		*Total*				
	2018^R^	2018^NR^	2018^R^	2018^NR^	2018^R^	2018^NR^	2018^R^	2018^NR^	2007^R^	2018^R^	2018^NR^	Increase (#|%)^a^	Reduction (#|%)^b^
C	3566	322	2061	312	1736	293	454	114	*1278*	*7817*	*1041*	6539 | 511%	6776 | 86.68%
prM	2651	364	2376	329	1787	168	659	89	*1530*	*7473*	*950*	5943 | 388%	6523 | 87.29%
E	2329	1074	5269	1533	2950	933	1724	543	*3845*	*12,272*	*4083*	8427 | 219%	8189 | 66.73%
NS1	2470	491	2190	488	1314	306	397	114	*1784*	*6371*	*1399*	4587 | 257%	4972 | 78.04%
NS2a	1982	411	1535	349	1012	207	334	97	*705*	*4863*	*1064*	4158 | 589%	3799 | 78.12%
NS2b	1978	155	1537	126	1019	87	259	38	*614*	*4793*	*406*	4179 | 680%	4387 | 91.53%
NS3	1976	404	1578	384	1204	309	276	92	*695*	*5034*	*1189*	4339 | 624%	3845 | 76.38%
NS4a	1949	141	1519	114	993	84	241	40	*523*	*4702*	*379*	4179 | 799%	4323 | 91.94%
NS4b	1952	193	1524	261	999	97	319	77	*602*	*4794*	*628*	4192 | 696%	4166 | 86.90%
NS5	2021	742	1995	749	1334	494	421	149	*828*	*5771*	*2134*	4943 | 596%	3637 | 63.02%
*Total*	*22,874*	*4297*	*21,584*	*5020*	*14,348*	*2978*	*5084*	*1353*	*12,404*	*63,890*	*13,648*	*51,486 | 415%*	*50,242 | 78.64%*

^R^Number of redundant sequences collected from the National Center for Biotechnology Information (NCBI) Taxonomy database in December 2007 [35] and April 2018

^NR^Number of non-redundant sequences after removal of duplicate sequences (full length and partial)

^a^Number and percentage of redundant sequences increase from 2007 [35] and 2018

^b^Number and percentage of sequence reduction for the 2018 dataset as a result of the removal of duplicate sequences; rounded to two decimal places

### Evolutionary diversity of DENV proteome

**Fig. 2:**
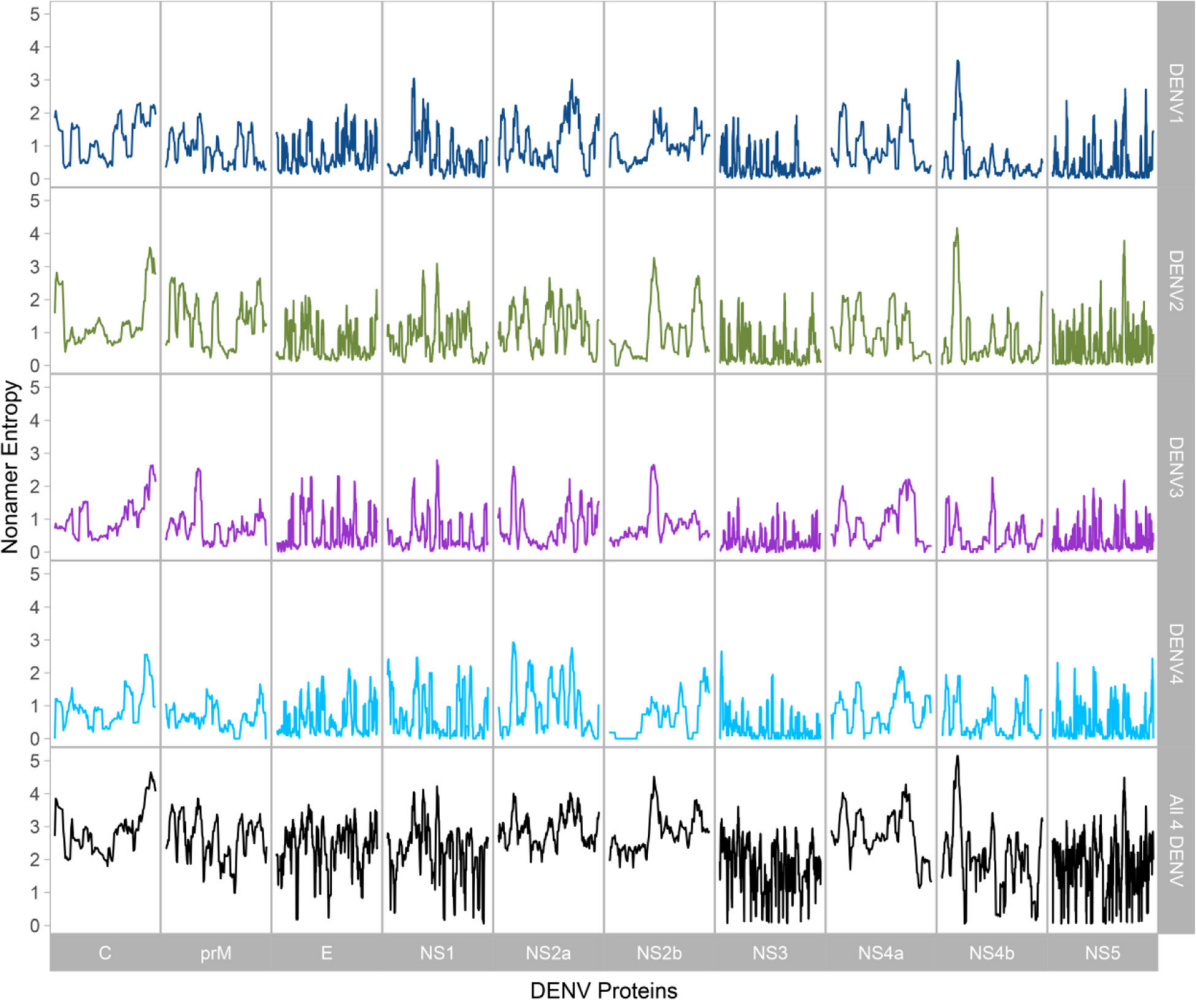
Sequence diversity of DENV proteomes, within (top four) and across (bottom) the four serotypes. The Shannon’s entropy values were computed from the alignments of DENV sequences using the tool AVANA, as described in the *Methods*. Centre, instead of starting positions were used herein for the plot (everywhere else, starting positions are used), and thus, the first and last four positions in the alignment of each protein were not assigned any peptide entropy value as they cannot be the centre of a nonamer

The variability of nonamer peptide sequences of each DENV serotype individually and all the four serotypes combined were studied by use of Shannon’s entropy (Fig. 2). A relatively high degree of intra-type sequence conservation was observed, with low entropy values, generally below 0.8, and numerous pockets of regions with entropy equal and close to zero, particularly in NS3, NS4b and NS5. The protein DENV2 C was the most diverse with an average peptide entropy of ~ 1.339, while the protein DENV4 NS3 was the most conserved with the lowest average entropy value of ~ 0.361. The absolute, maximum intra-type entropy values were: DENV1 NS4b_44–52_, ~ 3.585; DENV2 NS4b_42–50_, ~ 4.163; DENV3 NS1_170–178_, ~ 2.791; and DENV4 NS2a_33–41_, ~ 2.927. The difference in the entropy values between the proteins of the four types resulted in a marked increase in the peptide entropy across all DENVs. The combined entropy of all 4 DENV types had protein NS3 still as the most conserved, but with a much higher average entropy value (~ 1.777), while NS2a punctuated as the most diverse with the highest (~ 2.907) average entropy value. The maximum inter-type entropy value was 5.148, which was from NS4b_43–51_. Khan et al. [35] performed a similar analysis with DENV sequences (entropy analysis was done with a dataset earlier than the 2007, up to date as of 2005). The redundant data used herein increased by ~ 441.27%. In general, after a decade, there was an increase in the average minimum and maximum entropy values, however, the intra-type increase (~ 1.8 fold) was much higher than inter-type (~ 1.3 fold) (Additional file 1: Table S1). The serotypes that exhibited the minimum (DENV4) and maximum (DENV2) average intra-type entropy values remained the same between the two time points, however, the proteins changed; instead of NS4b, NS3 exhibited the minimum, while C, instead of prM, exhibited the maximum. Conversely, the proteins that exhibited the minimum (NS3) and maximum (NS2a) average inter-type entropy values remained the same between the two time points. Notably, the absolute maximum intra-type entropy values also increased from ~ 3.2 in DENV1 NS5 (2005 data) to 4.163 (2018 data), but in a different serotype and protein (DENV2 NS4b). The peak inter-type value also increased, from ~ 4 to 5.148, however, the protein (NS4b) and the localization remained the same.

### Identification of highly conserved, serotype-specific (HCSS) sequences

**Fig. 3:**
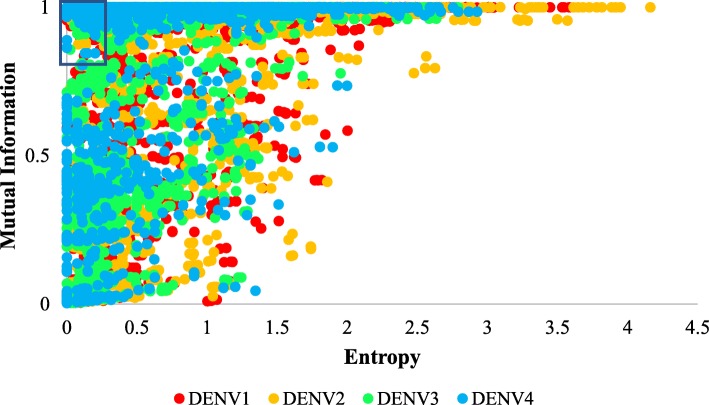
Scatter plot of entropy and mutual information (MI) values for all nonamer positions of each DENV serotype proteins. The boxed region (MI of > 0.8 and Entropy of < 0.25) is the selected cut-off threshold for identification of HCSS nonamers

A total of 2321 HCSS nonamers were identified with entropy of < 0.25 and MI > 0.8 (Table 2; Fig. 3): DENV1 (459 nonamers), DENV2 (465 nonamers), DENV3 (565 nonamers) and DENV4 (832 nonamers). Amongst these, DENV1 NS5 had the most number of such sequences (227 nonamers), while C had the least (only one nonamer). All HCSS nonamers were subsequently concatenated together if they overlapped by at least one amino acid, resulting in the number reduction to 337 HCSS sequences (Additional file 2: Table S2). Among these, 280 sequences were at least 10 amino acids long, with the maximum length of 53 amino acids, present in NS5 of DENV1.

**Table 2:** Number of highly conserved, serotype-specific (HCSS) nonamers

Protein / Serotype	DENV1	DENV2	DENV3	DENV4	*Total*
C	0	0	0	1	*1*
prM	5	1	9	11	*26*
E	11	65	81	149	*306*
NS1	32	29	77	88	*226*
NS2a	15	10	37	35	*97*
NS2b	1	15	3	44	*63*
NS3	110	158	87	223	*578*
NS4a	3	3	12	17	*35*
NS4b	55	41	39	60	*195*
NS5	227	143	220	204	*794*
*Total*	*459*	*465*	*565*	*832*	*2321*

**Fig. 4:**
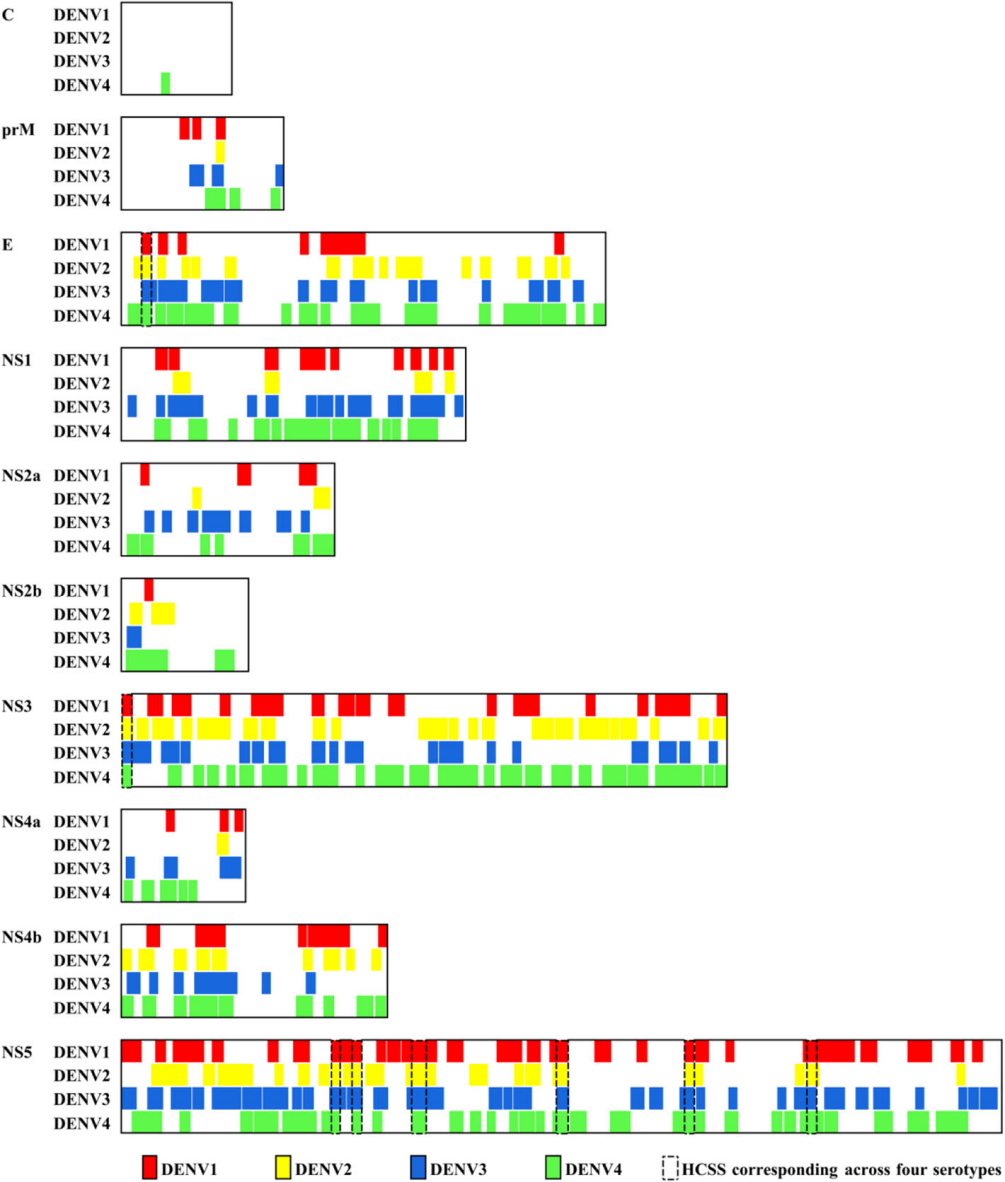
DENV proteome map of highly conserved, serotype-specific (HCSS) sequences. The width of the boxes corresponds to the length of the proteins. Coloured boxes represent the location of the HCSS sequences within each serotype: red, DENV1; yellow, DENV2; blue, DENV3; and green, DENV4. The dotted rectangular boxes represent regions of the proteome where distinct HCSS sequences corresponded across the four serotypes

A map of the HCSS sequences within the DENV proteomes is illustrated in Fig. 4. The proteins DENV4 NS3 and DENV2 prM were the most (~ 69.95%) and least (~ 5.42%) packed with HCSS sequences (defined as contiguous length of the HCSS sequences over the length of the protein). Notably, there were marked differences in the correspondence and the relative degree of MI and entropy values (Fig. 3) for the HCSS sequences of each protein between the four serotypes. Eight HCSS sequence positions corresponded across the four serotypes, with a distinct HCSS sequence for each serotype (Additional file 3: Table S3). The average MI and entropy values for these eight positions were nearly 1 and < 0.184, respectively. There were, on average, two amino acid mutations between the distinct HCSS sequences of the serotypes. HCSS sequence positions with correspondence to three or two serotypes were also observed. As many as 104 HCSS sequences showed no correspondence (i.e. only observed in a single serotype). Analysis of four HCSS nonamer positions that had a maximum MI of 1 and low entropy (0 to 0.23), which included three positions with no correspondence and one between two serotypes, exhibited a larger number of amino acid substitutions (Table 3; Additional file 4: Table S4). Positions with no correspondence, on average, showed one amino acid difference between the HCSS sequence and its variants from the same serotype (reflecting the low entropy selection criteria for the HCSS), and a larger (on average, four) amino acid difference to variants of the other serotypes. Similar, and possibly higher, amino acid (aa) difference was observed when correspondence was not across the four serotypes; average of seven aa difference to variants, including the HCSS, for NS2a_202–210_, which showed correspondence to two serotypes.

**Table 3:** Nonamer positions depicting amino acid differences between an HCSS nonamer and the corresponding variants, within and between the serotypes. Only positions of mutual information value of 1 and low entropy values are shown. HCSS nonamers are shown in yellow, and one is arbitrarily chosen as the reference when more than one corresponding HCSS nonamers are present. Data for two additional positions are shown in Additional file 4: Table S4

Protein | Entropy Value	NS1 | 0.12			NS2a | 0.11		
HCSS Reference	**146**	**NRAWNSLEV**	**154**	**202**	**LNPTAIFLT**	**210**
DENV1	146	Q....IW..	154	202	CK.LTM..I	210
	146	Q....VW..	154	202	CK.LPM..I	210
	146	L....IW..	154	202	CK.LTML.I	210
	146	H....IW..	154	202	CK.LTM.FI	210
	146	Q....IWK.	154	202	CK.LTMY.I	210
	146	Q..S.IW..	154	202	CK.L.M..I	210
	146	Q....IW.G	154	202	CK.L.ML.I	210
	146	Q....I...	154	202	CK.LTM..V	210
	146	Q...TIW..	154	202	CK.STM..I	210
				202	C..LTM..I	210
				202	SK.LTM..I	210
				202	CK.LTMYFI	210
				202	CKTLTM..I	210
DENV2	146	.........	154	202		210
	146	S........	154	202	I	210
	146	.......K.	154	202	S	210
	146	.......KG	154	202	L	210
	146	..T.D....	154	202	YF	210
	146	.......KL	154			
DENV3	146	S....VW..	154	202	VP.LPL.IF	210
	146	A....VW..	154	202	VP.LPLLIF	210
	146	L....VW..	154	202	IP.LPL.IF	210
	146	S..L.VW..	154	202	.P.LPL.IF	210
				202	VQ.LPL.IF	210
				202	VS.LPL.IF	210
				202	VP.SPL.IF	210
				202	VPSLPL.IF	210
				202	AQ.LPL.IF	210
DENV4	146	R........	154	202	AQALPVY.M	210
	146	R....F...	154			
	146	R.....F..	154			
	146	R....FF..	154			

### Functional analysis of the HCSS sequences

Less than half of the HCSS sequences (153 of 337) were predicted to be of functional relevance (Additional file 5: Table S5). Protein E corresponded to three functional domains and motifs, namely central and dimerization domain, immunoglobulin-like domain III and stem/anchor domain. Whilst, HCSS from NS3 were predicted to be required for peptidase S7, p-loop containing nucleosidetriphosphate hydrolases, DEAD domain and helicase domain. HCSS of NS5 corresponded to RNA dependent RNA polymerase (RdRp) domain, while one HCSS of the prM was predicted as a propeptide.

### Predicted T-cell epitopes within the HCSS sequences

**Fig. 5:**
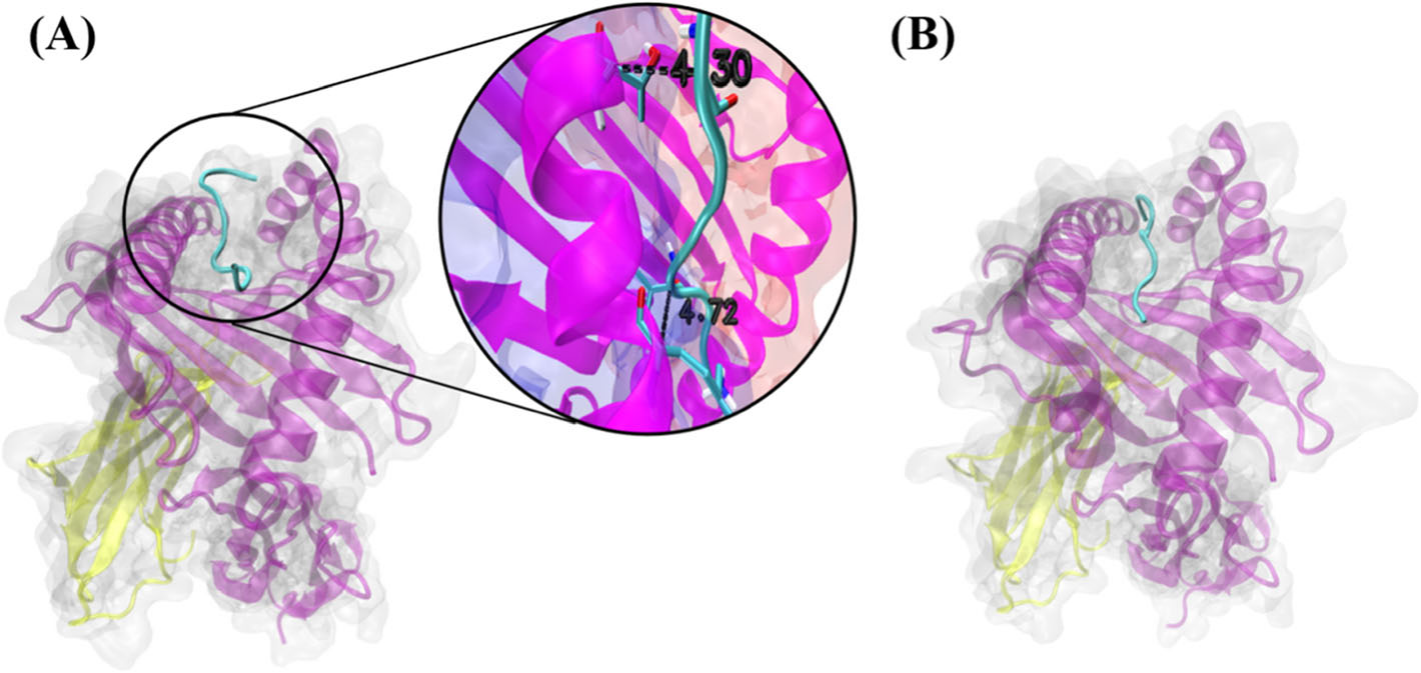
Visualization of epitope-receptor binding by use of ClusPro molecular docking. Panel A: a docked complex of a representative putative epitope (DENV4 NS3 _335_YQGKTVWFV_363_) and HLA-A2*0201 receptor (PDB ID: 2GIT). Panel B: docked control, known peptide-HLA complex (PDB ID: 3SPV). Peptide in either complex is represented by a cyan ‘New Cartoon’ structure, while HLA receptor is represented by a silver transparent ‘QuickSurf’ and ‘New Cartoon’ (chain α: purple; chain β: yellow). The inset in panel A shows two interactions between the epitope and the HLA receptor (chain α1: blue ‘QuickSurf’ background; chain α2: red ‘QuickSurf’ background) within the cut-off distance of 5.0 Å, which are 4.30 Å and 4.72 Å

A total of 154 distinct putative epitopes, restricted against HLA-A, -B and -DR supertypes, were predicted within the 337 HCSS sequences. DENV4 had the highest number of predicted epitopes (60), representing ~ 39% of the total epitopes predicted; followed by DENV3 (30; ~ 19.48%), DENV2 (33; ~ 21.43%) and DENV1 (31; ~ 20.13%) (Table 4). Epitope receptor docking of the DENV4 NS3 peptide _335_YQGKTVWFV_363_ against the receptor of HLA-A2*0201, showed potential binding (lowest energy: − 887.8 kcal/mol), relative to the docking of a control, known peptide-HLA complex (lowest energy: − 979.4 kcal/mol) (Fig. 5). This further supported the reliability of the sequence-based prediction employed.

**Table 4:** HLA-A, -B and -DR supertype-restricted T-cell epitopes, predicted for HCSS nonamers, summarised according to DENV protein and serotypes

Protein	Serotype	MHC Class I								MHC Class II			Total^a^	Non-redundant Total^a^	Total^b^	Non-redundant Total^a^
		HLA A supertypes			HLA B supertypes					HLA DR supertypes						
		A1	A2	A3	B7	B27	B44	B58	B62	Main DR	DR4	DRB3				
prM	DENV3	–	–	–	–	–	–	–	–	–	1	–	*1*	*1*	*5*	*2*
	DENV4	1	–	–	–	–	–	–	1	1	1	–	*4*	*1*		
E	DENV1	–	–	–	–	–	–	1	–	–	–	–	*1*	*1*	*25*	*24*
	DENV2	–	1	2	–	1	1	–	–	–	1	–	*6*	*5*		
	DENV3	–	–	1	–	–	1	1	–	–	3	–	*6*	*6*		
	DENV4	–	2	2	1	–	3	3	–	1	–	–	*12*	*12*		
NS1	DENV1	–	–	–	–	–	–	–	–	1	1	–	*2*	*1*	*14*	*13*
	DENV2	–	–	–	–	–	–	2	–	–	–	–	*2*	*2*		
	DENV3	–	–	1	–	–	1	1	–	–	–	–	*3*	*3*		
	DENV4	–	1	–	1	1	1	2	1	–	–	–	*7*	*7*		
NS2a	DENV1	–	–	–	–	–	1	2	–	–	–	–	*3*	*3*	*23*	*16*
	DENV3	–	–	1	–	1	–	1	–	3	3	–	*9*	*7*		
	DENV4	–	–	–	2	1	–	–	–	3	3	2	*11*	*6*		
NS2b	DENV2	–	–	1	–	–	–	–	–	2	–	–	*3*	*3*	*11*	*8*
	DENV4	1	2	–	–	–	–	1	1	2	1	–	*8*	*5*		
NS3	DENV1	2	–	–	–	–	–	2	2	1	1	2	*10*	*6*	*36*	*28*
	DENV2	1	1	2	1	–	5	–	1	1	–	–	*12*	*10*		
	DENV3	1	–	–	1	–	–	–	–	–	–	–	*2*	*2*		
	DENV4	1	2	–	3	–	1	2	1	–	1	1	*12*	*10*		
NS4a	DENV1	–	–	–	1	–	–	–	–	–	–	–	*1*	*1*	*5*	*5*
	DENV2	–	–	–	1	–	–	–	–	–	–	–	*1*	*1*		
	DENV3	–	–	–	–	–	1	–	–	–	–	–	*1*	*1*		
	DENV4	–	1	–	–	–	–	–	–	–	–	1	*2*	*2*		
NS4b	DENV1	–	1	–	–	–	–	1	–	1	1	1	*5*	*3*	*23*	*13*
	DENV2	–	1	–	1	1	–	2	2	2	1	–	*10*	*5*		
	DENV3	–	1	–	–	–	–	–	–	–	–	–	*1*	*1*		
	DENV4	–	–	2	–	–	–	1	1	1	1	1	*7*	*4*		
NS5	DENV1	1	–	3	2	1	2	5	2	2	3	2	*23*	*16*	*61*	*45*
	DENV2	–	1	2	–	–	1	–	–	1	1	2	*8*	*7*		
	DENV3	1	1	2	–	1	1	–	–	1	2	2	*11*	*9*		
	DENV4	1	1	2	1	1	2	5	3	1	1	1	*19*	*13*		
Total^c^		*10*	*16*	*21*	*15*	*8*	*21*	*32*	*15*	*24*	*26*	*15*				*154*
Gene level total^c^		*47*		*91*						*65*						

^a^Total number of predicted epitopes for each serotype with respect to each protein

^b^Total number of predicted epitopes for each protein

^c^Total number of predicted epitopes for each supertype

The 154 predicted epitopes represented a total of 47 HLA-A (redundant listing: 10 for A1; 16 for A2; 21 for A3) and 91 HLA-B (redundant listing: 15 for B7; 8 for B27; 21 for B44; 32 for B58; 15 for B62) supertype-restricted T-cell epitopes (Table 4; Additional file 6: Table S6). Similarly, as many as 65 HLA Class II (HLA-DR; redundant listing: 24 for Main DR; 26 for DR4; 15 for DRB3) supertype-restricted T-cell epitopes were predicted (Table 4; Additional file 6: Table S6). In general, NS5 was enriched with the most number of supertype-restricted epitopes (~ 29.22%; 45 non-redundant epitopes), followed by NS3 (~ 18.18%; 28 non-redundant epitopes), whereas prM had the least with only 2 epitopes (~ 3.7%) restricted by supertypes.

There were 31 predicted supertype-restricted T-cell epitopes that appeared to be promiscuous to more than one supertype, with 11 spanning both class I and II (Additional file 6: Table S6). The promiscuity of these 31 putative epitopes extended to inter-supertype (17; restricted for at least two supertypes of the same HLA gene), inter-HLA gene (seven; restricted for at least two supertypes of distinct HLA gene), or inter-HLA class (seven; restricted for at least two supertypes of different HLA class).

### Matching of experimentally validated and predicted T-cell epitopes

**Fig. 6:**
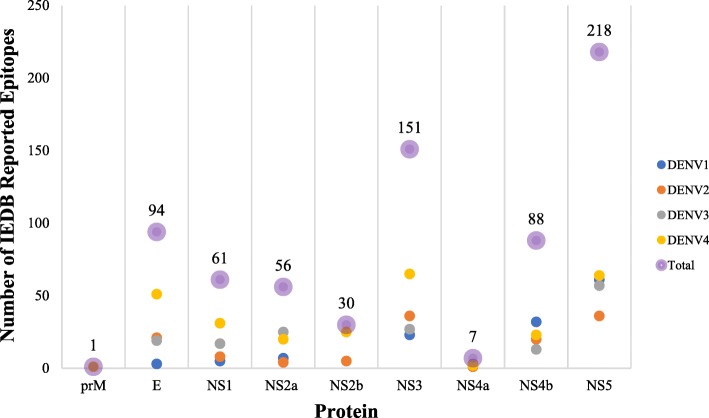
IEDB reported DENV T cell epitopes/ligands in human that completely matched HCSS sequences

The HCSS appeared highly immunogenic, as 198 of the sequences included 706 experimentally validated DENV T-cell epitopes reported and readily available in the public repository, IEDB (Fig. 6; Additional file 7: Table S7). Allele HLA-A*11:01 was most well studied and HLA-A*29:02, HLA-A*69:01, HLA-B*15:17, HLA-B*15:42, HLA-B*45:06, HLA-B*48:01, HLA-B*83:01 and HLA-C*04:01 were the least studied. The protein NS5 was most packed with the IEDB-reported immunogenic epitopes across the DENV serotype (218 epitopes, ~ 30.87%). The DENV4 proteome was reported with the most number of epitopes (282 epitopes, ~ 39.94%). Out of 198 HCSS sequences containing experimentally validated epitopes, only 121 appeared to be restricted by at least two representative alleles of a given supertype studied. Amongst the 198, 87 (149 distinct epitope sequences) matched the predicted nonamer epitopes (**representative Protein E in** Table 5; Additional file 8: Table S8). Of these 87, 37 were clusters of immunological hotspots (17 intra-supertype regions; three inter-supertype regions; 11 inter-HLA gene regions and six inter-HLA class regions), with length ranging from 10 to 46 amino acids. In brief, DENV1 NS5 comprised of the most hotspot (5) regions. Among these, three hotspots contained epitopes that were potentially intra-supertype promiscuous.

**Table 5:** Reported epitopes that matched the predicted epitopes of HCSS sequences for structural protein E. Full data for other DENV proteins are provided in Additional file 8: Table S8

Protein	Serotype	Matched Epitopes (Starting Position | Ending Position)	HCSS Sequence	Supertype Predicted	Supertype Reported (IEDB
E	DENV1	204|212	204 KSWLVHKQWFKTAHAKKQE 249	B58	B58: HLA-B*57:01, HLA-B*58:01
	DENV2	210|218, 213|221	210 RQWFLDLPLPWLPG 223^#^	A2	A2: HLA-A*02:06, HLA-A*02:01, HLA-A*02:17
				B27	B27: HLA-B*27:05, HLA-B*48:01
				B44	B44: HLA-B*40:01
		238|246	237 LVTFKNPHAKKQDVVVLGSQE 257	A3	A3: HLA-A*03:01, HLA-A*11:01, HLA-A*68:01
		296|305, 297|305	281 GHLKCRLRMDKLQLKGMSYSMCTGKFK 307^$^	A3	A3: HLA-A*03:01, HLA-A*11:01, HLA-A*68:01
	DENV3	204|212, 211|220, 212|220	204 KAWMVHRQWFFDLPLPW 220^#^	A24	A24: HLA-A*23:01, HLA-A*24:02
				B44	B44: HLA-B*44:03
				B58	B58: HLA-B*57:01, HLA-B*58:01
		238|246	234 KELLVTFKNAHAKKQ 248	A3	A3: HLA-A*03:01, HLA-A*11:01, HLA-A*68:01
		313|321	306 FVLKKEVSETQHGTILI 322	B44	B44: HLA-B*40:01, HLA-B*44:03
	DENV4	51|59, 51|60	47 KTTAKEVALLRTYCIEA 63^$^	B44	B44: HLA-B*44:02, HLA-B*44:03
		65|73, 82|90	65 ISNITTATRCPTQGEPYLKEEQDQQYICRR 94^#^	A3	A3: HLA-A*31:01
				B44	B44: HLA-B*40:01, HLA-B*44:03
		164|173, 165|173	164 ITPRSPSVEV 173^#^	A2	A2: HLA-A*68:02
				B7	B7: HLA-B*07:02, HLA-B*51:01
		204|212	204 KTWLVHKQWF 213	B58	B58: HLA-B*57:01, HLA-B*58:01
		237|246, 238|246, 238|247	235 ERMVTFKVPHAKRQDVTVLGSQEGAMHSAL 264^$^	A3	A3: HLA-A*03:01, HLA-A*11:01, HLA-A*68:01
		313|321	290 EKLRIKGMSYTMCSGKFSIDKEMAETQHGTTVV 322	B44	B44: HLA-B*40:01, HLA-B*44:03
		412|420	391 WFRKGSSIGKMFESTYRGAKRMAILGETAWDFGSVGGL 428	B58	B58: HLA-B*57:01, HLA-B*58:01
		445|453	430 TSLGKAVHQVFGSVYTTMFGGVSWM 454	B58	B58: HLA-B*57:01, HLA-B*58:01

Highly conserved, serotype-specific (HCSS) sequences with at least two matched (reported and predicted) epitopes (hotspot) that show ^$^intra-supertype restriction (epitope that is restricted by only one supertype of HLA gene; i.e. A1 supertype-restricted epitope) and ^#^inter-HLA gene supertype restriction (epitope that is restricted by at least two supertypes of different HLA gene; i.e. A2 and B7 supertype-restricted epitope)

## Discussion

The conserved epitope paradigm has been a major focus for identification of vaccine targets that address the diversity of pathogens [35, 71–74]. Sequences with extended conservation across different groups of a pathogen, such as influenza A virus (IAV) subtypes, have been proposed as universal vaccine candidates [72, 75]. The copiousness of such sequences decreases as pathogen sequence diversity increases; as such, they are often limited in number and length for pathogens that exhibit reasonable sequence diversity, such as in DENV, IAV, and human immunodeficiency viruses (HIV)-1 proteomes. This is further exacerbated when the conservation is extended to other family members, a consideration given the possibility of APLs as a result of similar genomic architecture between family members [29, 76]. For example, DENV and HIV-1 had 44 and 78 highly conserved sequences each, respectively, however, only 27 and 74 were conserved across majority of the family members [77]. The remaining were either not present in the family members or were represented with conservation that fluctuated from low to high between the members.

Consequently, Khan et al. [35] proposed a focus on conserved sequences that are species specific to avoid the issue of variant APLs from family members, where the conserved epitope is not highly represented. Inadvertently, this further reduces the number of usable conserved sequences for vaccine design. Even a highly conserved pathogen with a larger number of conserved sequences, may end up with a limited number that are species specific. For example, West Nile virus (WNV) had 88 sequences (~ 34% of the WNV proteome) that were highly conserved with 100% representation within the reported viral sequences, however, only 21 were species-specific [77]. This may be mitigated by restricting the specificity to a species sub-group level (if pan-subgroup specificity is not essential), such as specific at DENV serotype level rather than DENV species. This can provide for a large number of conserved sequences, of longer length, possibly capturing regions of B-cell epitopes, and minimise cross-reactivity between the sub-groups. The HCSS sequences identified herein are such sequences for DENV that serve as an alternative strategy to pan-DENV sequences in limiting variant peptides.

The large number of DENV viral protein sequences available in public repositories offered a corpus of data for the study of HCSS sequences. The data provided for a broad temporal (30 years) and spatial (> 100 countries) coverage. The majority of the sequences, however, turned out to be duplicates, with only ~ 21.36% non-redundant sequences across the DENV1–4 serotypes, and at a similar level for the individual proteins, except for E and NS5. The redundancy reflected sampling bias to identical or highly similar circulating DENV isolates sequenced from various geographical localities. Although the redundancy may be an indication of the incidence of the corresponding DENV isolates in nature, we minimised bias by using non-redundant sequences for subsequent analyses.

Entropy analysis enabled study of the evolutionary diversity within and between the DENV serotypes. Overall, DENV sequences were highly conserved within the serotypes; however, there was a marked increase in the combined peptide entropy between the four DENV serotypes. This reflected relatively low degree of sequence conservation across the DENV1–4 proteomes (Fig. 2). Khan et al. [35] performed a similar analysis with DENV sequences. After a decade, there was a general increase in the entropy values, within and between the dengue serotypes, indicating a greater diversity spectrum. The increase in the peak diversity of dengue virus protein sequences (from ~ 4 to ~ 5.148) brings it a notch closer to that of influenza A viruses (~ 6.0; 2006 data) [72], but still distance from HIV-1 (~ 9.0; clade B; 2008 data) [74].

Mutual information (MI) together with entropy were used to identify HCSS nonamers. MI is a method for identifying amino acid sites that distinguish specific sets of protein sequences, by comparative analysis of matched alignments, such a co-alignment of DENV1 against the other serotypes. Entropy is a measure of a disorder, and allows quantification of sequence conservation. MI analysis had been previously utilised by Miotto et al. [46, 47] for large-scale identification of human-to-human transmissibility factors in proteins of influenza A, with a selection threshold of MI > 0.4. The HCSS nonamers were identified from the proteome dataset by use of the restricting parameters of low entropy at < 0.25 within the serotype of interest and high MI of > 0.8 between the serotypes, signifying a strong association of the amino acid variants distribution (Fig. 3). This resulted in a 459 to 832 nonamers, covering an average length of ~ 39.99% (DENV1: ~ 32.51%; DENV2: ~ 32.23%; DENV3: ~ 42.18%; DENV4: ~ 53.03%) of the DENV proteomes (~ 3390 amino acids). Although higher MI (ideally 1, as the highest point of distinction between the serotype of interest and the other serotype datasets) and lower entropy (ideally 0) are desired, the fraction of the proteome represented by HCSS would inversely reduce. Thus, the defined MI threshold herein aimed to balance the number and the specificity of the resulting sequences. DENV4 was packed with the most number of HCSS nonamers (832 nonamers, ~ 35.85%), while DENV1 was least packed (459 nonamers; ~ 19.78%) (Table 2). This is in agreement with phylogenetic analysis of the four serotypes, with DENV4 generally the most distinct and highly conserved [78].

It is noteworthy that NS5, among the highly conserved proteins of each serotype [14, 79], had the highest total number of HCSS nonamers (794 nonamers, ~ 34.21%) and also the single longest HCSS sequence (53aa). This was followed by NS3 (the most conserved protein of each serotype [14]; (578 nonamers, ~ 24.90%), which also was the most packed with HCSS over the protein length) and Envelope (among the diverse proteins of each serotype) (306 nonamers; ~ 13.18%). Although the functional role of the large majority of HCSS is unknown, less than half were predicted to be functionally important. NS3 and NS5 have an important role in capping, methylation and viral replication [79–82]. Viral replication requires protease and helicase activities, facilitated by NS3 peptidase S7 and helicase domains [83]. The protein E is the main antigenic, surface-exposed determinant on the virion [84]. The dimerization domain II contributes to virus-mediated membrane fusion by interacting with a cellular receptor [85, 86]. The C-terminal of protein E domain III is anchored to helices and transmembrane helices by the linkage of disulfide bridges, while the N-terminal, which is formed by β-strands, is folded into an immunoglobulin-like domain that is important in receptor recognition. The HCSS within these proteins are likely robust given the important functional and structural roles, and merit investigation as vaccine targets.

There is evidence that the HCSS sequences are immune-relevant, supported by sequence-based, structure-based and experimental assessments. As many as 706 DENV reported T-cell epitopes and/or HLA ligands in human completely matched (substring matches excluded) more than half (198 of the 337; ~ 58.58%) of the HCSS sequences. Numerous (~ 35.80%; 121/337) of the HCSS sequences showed proclivity for restriction to at least two representative alleles of a supertype, and thus are potentially promiscuous epitopes. Moreover, among the 337 HCSS sequences, as many as 154 were predicted to be promiscuous for representative alleles of 11 major HLA class I supertypes and three class II DR supertypes. The supertype restriction provides for a broad coverage of the human population, with multiple (19) HCSS exhibiting enhanced promiscuity across different supertypes within and between HLA genes and even between HLA classes. Such a higher degree of promiscuity has been reported by others [87–89] and they are better candidates for vaccine design given the extended population coverage. Many (87) of the 198 HCSS sequences that matched to reported T-cell epitopes/ligands in human also matched the predicted epitopes, supporting the validity of the predictions.

A total of the 37 HCSS sequences were both matching the predicted and the reported epitopes/HLA ligands, and were also clustered as immunological hotspots (Additional file 8: Table S8). These hotspots are noteworthy as preferred targets for vaccine development because putative promiscuous epitopes are in a clustered region. Ideally, inter-HLA class supertype hotspots are attractive because besides providing a broad population coverage, they are also relevant to both CD8^+^ and CD4^+^ cellular T-cells immune response [58]. Highest number of hotspot regions were observed in DENV1 NS5, with restrictions for HLA-A1, -A3, -B7, -B44, -B58, and -B62 supertypes. According to several studies of Weiskopf et al., CD8^+^ T-cells immune response are predominantly present in NS proteins, specifically NS4b and NS5, of DENV2, thereby potentially important for immunodominance response of the serotype-specific sequences [23, 90, 91], while the immunodominance patterns for DENV3 are mainly towards the structural proteins, specifically M, despite the immune response elicited by both structural and non-structural proteins.

The HCSS sequences represent, on average, ~ 40% of the proteome length of each of the serotypes (Fig. 4). This is more than double of the proteome length represented by pan-DENV sequences [35]. The larger coverage offers a multitude of choices for selection of sequences as vaccine targets. Also, HCSS sequences offer a larger, single contiguous length (10–53aa) compared to pan-DENV sequences (9–22aa), allowing for consideration of even conformational (such as neutralizing) antibody epitopes, which has been shown to be an important correlate of protection [24, 92, 93]. This is particularly so given that numerous (59) HCSS sequences are present in the structural proteins, in contrast to two for pan-DENV sequences. HCSS sequences are observed in all the three structural proteins and predominantly (48) in the protein E, in contrast to only two pan-DENV sequences in the protein E. The envelope HCSS sequences are also longer, 10 of them are more than 20 amino acids with the longest 38 amino acids, nearly double of each of the two pan-DENV sequences of E (10-15aa). The pan-DENV sequences were absent in C, prM, NS2a, and NS2b, whereas the HCSS sequences are present in all the DENV proteins. Clearly, the HCSS sequences offer a larger choice of sequences for vaccine target selection.

The sequence diversity between the proteins of the four DENV serotypes is among the key issues in the development of a tetravalent vaccine that provides an effective protection against each of the serotypes [31, 35]. The amino acid variability within and between serotypes can range from ~ 1–21% to ~ 14–67%, respectively [14]. Amino acid differences between recognised T-cell epitopes in the case of sequential heterologous infection, can alter the outcome of the response, from being protective to pathogenic [26–30]. A focus on the conservation spectrums of sequence diversity, pan-DENV serotypes to serotype-specific, may represent an avenue to subvert the pathogenic effects. Towards this, the former approach aims to limit the number of possible cross-reactive epitope variants in the population, relevant to a given memory response, while the latter aims to limit the cross-reactivity between the serotypes to favour a serotype-specific response. The work by Khan et al. [35] was an attempt to report on the former; the HCSS sequences reported herein represent the latter approach. HCSS sequences showed significant amino acid difference to all the variants across the serotypes with increasing MI value, which also resulted in a decreased occurrence of corresponding HCSS sequences between the serotypes.

There is evidence that both neutralizing antibody and specific T-cell responses are required [12, 92–94] for protection against dengue. The incorporation of supertype-restricted T-cell epitopes within DENV vaccine candidates may improve vaccine efficacy by providing for a robust long-lived immunity through cytostatic and/or cytotoxic effects, as well as the wide population coverage [95]. For tetravalent formulations, HCSS sequences may be evaluated for inclusion, besides the consideration of pan-DENV sequences. Among the 337 HCSS sequences identified herein, the following maybe utilised as prioritisation criteria: i) high MI value ii) low intra-serotype entropy, iii) no or little correspondence between serotypes, iv) immune-relevant, v) supertype-restricted, vi) extended HLA promiscuity, vii) a hotspot, and viii) of longer-length, increasing possibility of B-cell epitope(s) within (top 20 HCSS sorted according to this criteria are provided in Table 6). Further investigations are needed to validate the immunogenicity and the protective role of the HCSS sequences in human subjects.

**Table 6:** Top 20 candidate HCSS sequence, sorted according to prioritisation criteria

Protein	Serotype	HCSS Sequence	MI Value	Entropy Value	Number of Epitopes	Number of Supertype Restrictions	Extended HLA Promiscuity	Length of HCSS Sequence
NS2a	DENV4	196 TALILGAQALPVYLMTLMKGAS 217	1	0.072	3	2	Yes	22
NS2b	DENV4	96 TLLVKLALITVSGLYPLAIP 115	1	0.092	5	3	No	20
NS4b	DENV4	241 WNTTIAVSTANIFRGSY 257	1	0.168	2	2	Yes	17
NS1	DENV4	34 FQPESPARLASAILNAH 50	1	0.186	2	1	No	17
NS2b	DENV2	31 PLVAGGLLTVCYVLTGRSADLELE 54	1	0.199	2	1	No	24
NS2a	DENV1	119 SLEELGDGLAMGIM 132	1	0.219	2	1	No	14
NS2b	DENV4	5 NEGIMAVGLVSLLGSALLKNDVPLAGPMVAGGLLLAAYVMSGS 47	0.999	0.052	8	2	Yes	43
NS5	DENV1	484 LEFEALGFMNEDHWFSR 500	0.999	0.052	2	1	No	17
NS5	DENV1	756 GKSYAQMWQLMYFHRRD 772	0.999	0.064	5	3	No	17
NS5	DENV1	848 CGSLIGLTARATWA 861	0.999	0.066	2	1	No	14
NS3	DENV2	32 GYSQIGAGVYKEGTFHTMWHVT 53	0.999	0.133	4	4	No	22
NS3	DENV4	324 DPFPQSNSPIEDIEREIPERSWNTGFDWITDYQGKTVWFVP 364	0.999	0.136	3	2	No	41
NS3	DENV2	78 SYGGGWKLEGEWKEGEEVQVLALEPGKNPRAVQT 111	0.999	0.189	2	1	No	34
E	DENV4	164 ITPRSPSVEV 173	0.998	0.155	2	2	No	10
E	DENV4	47 KTTAKEVALLRTYCIEA 63	0.997	0.108	2	2	No	17
E	DENV4	65 ISNITTATRCPTQGEPYLKEEQDQQYICRR 94	0.997	0.168	2	2	No	30
E	DENV4	235 ERMVTFKVPHAKRQDVTVLGSQEGAMHSAL 264	0.997	0.185	3	1	No	30
NS5	DENV1	215 THEMYWVSCGTGNIVSAVNMTSRMLLNRFTM 245	0.996	0.090	2	1	No	31
NS4b	DENV2	207 TQVLMMRTTWALCEALT 223	0.996	0.168	3	2	No	17
NS3	DENV1	546 WLSYKVASEGFQYSDRRWCFDGERNNQVLEENMDVE 581	0.996	0.178	2	1	No	36

## Conclusion

This work provides a catalogue of HCSS sequences in the DENV proteome, as candidates for vaccine target selection. The methodology described herein provides a framework for similar application to other pathogens, where sub-group-specific immune response maybe desired, such as other flaviviruses and influenza A virus.

## Supplementary information

**Additional file 1:** **Table 1.** Comparison of intra- and inter-serotype entropy values between the 2005 (Khan et al., 2008) and 2018 datasets (this study).

**Additional file 2:** **Table 2.** Highly conserved, serotype-specific (HCSS) sequences

**Additional file 3:** **Table 3.** Highly conserved, serotype-specific (HCSS) sequences that corresponded across the four serotypes

**Additional file 4:** **Table 4.** Nonamer positions depicting amino acid differences between an HCSS nonamer and the corresponding variants, within and between the serotypes. Only positions of mutual information value of 1 and low entropy values are shown. HCSS nonamers are shown in yellow, and one is arbitrarily chosen as the reference when more than one corresponding HCSS nonamers are present. A) The variant amino acids are not shown, instead the number of such variants and the number of amino acid differences are indicated. B) All the variants and the amino acid differences are shown.

**Additional file 5:** **Table 5.** Functional analysis of the highly conserved, serotype specific (HCSS) sequences by use of Pfam (supporting ID in column F) and the Conserved Domains Database (CDD; supporting ID in column E). Sequences without reported functional correlations are denoted with '-'. Sequences with two different functional domains and motifs from Pfam and CDD are denoted with ^*^ and ^#^, respectively (column D).

**Additional file 6:** **Table 6.** HLA-A, -B and -DR supertype-restricted T-cell epitopes, predicted for HCSS nonamers, summarised according to DENV protein and serotypes

**Additional file 7:** **Table 7.** IEDB reported DENV T cell epitopes/ligands in human that completely matched HCSS sequences

**Additional file 8:** **Table 8.** Reported epitopes that matched predicted epitopes of HCSS sequences

## Data Availability

All the data that support the findings of this study are available in the supplementary materials.

## Abbreviations

APL: Altered peptide ligand
AVANA: Antigenic variability analyser tool
BLAST: Basic local alignment search tool
C: Capsid protein
CTL: Cytotoxic T lymphocytes
DENV: Dengue virus
DHF: Dengue hemorrhagic fever
DSS: Dengue shock syndrome
E: Envelope protein
HCSS: Highly conserved, serotype-specific
HLA: Human leukocyte antigen
IAV: Influenza A virus
IEDB: Immune Epitope Database and Analysis Resource
IFN: Interferon
MAFFT: Multiple alignment using Fast Fourier Transform
MHC: Major histocompatibility complex
MI: Mutual information
NCBI: National Center for Biotechnology Information
NS: Non-structural
prM/M: Precursor membrane/membrane protein
TCR: T-cell receptor
WNV: West Nile virus
