# Correction to: Identification of highly conserved, serotype-specific dengue virus sequences: implications for vaccine design

**DOI:** 10.1186/s12864-021-07444-1

**Published:** 2021-03-26

**Authors:** Li Chuin Chong, Asif M. Khan

**Affiliations:** Centre for Bioinformatics, School of Data Sciences, Perdana University, Jalan MAEPS Perdana, 43400 Serdang, Selangor Darul Ehsan Malaysia

**Correction to: BMC Genomics 20, 921 (2019)**

**https://doi.org/10.1186/s12864-019-6311-z**

Following publication of the original article [1], the authors noted that some of the changes suggested during the proofing stage were missed. The authors highlight these below and includes a few additional changes for clarity:

1) In the background part of the Abstract section:

The published version of the sentence: “The former aims to limit the number of possible cross-reactive epitope variants in the population, while the latter aims to limit the cross-reactivity between the serotypes to favour a serotype-specific response.” should have been as follows: “The former pan-DENV sequence approach aims to limit the number of possible cross-reactive epitope variants in the population, while the current HCSS sequence approach aims to limit the cross-reactivity between the serotypes to favour a serotype-specific response.”

2) In the result part of the Abstract section:

The published version of the sentence: “Concatenating these resulted in a total of 337 HCSS sequences. DENV4 had the most number of HCSS nonamers; NS5, NS3 and E proteins had among the highest, with none in the C and only one in prM.” should have been as follows: “DENV4 had the most number of HCSS nonamers; NS5, NS3 and E proteins had among the highest across the serotypes, with the lowest in the C. Concatenating the 2,321 nonamers resulted in a total of 337 HCSS sequences.”

3) In the result part of the Abstract section:

The published version of the sentence: “HCSS sequences of a given serotype showed significant amino acid difference to all the variants of the other serotypes, supporting the notion of serotype-specificity.” should have been as follows: “Observation of select HCSS sequences of a given serotype showed significant amino acid difference to the variants of the other serotypes, supporting the notion of serotype-specificity.”

4) In the Introduction section:

The published version of the sentence: “The pan-DENV sequences may be of utility in the design of tetravalent vaccine to avoid regions of T-cell immunity that are highly variable across the four serotypes, except when they are serotype-specific [33, 36].” should have been as follows: “The pan-DENV sequences may be of utility in the design of a vaccine against the four serotypes to avoid regions of T-cell immunity that are highly variable across the serotypes, except perhaps when they are serotype-specific [33, 36].”

5) In the Method section:

The published version of the sentence: “The blast parameters (E-value less than 0.05) were used to evaluate the significance of the hits and select the sequences for each serotype protein.” should have been as follows: “The blast parameters (E-value less than 0.05, among others) were used to evaluate the significance of the hits and select the sequences for each serotype protein.”

6) In Table 3:

The published version of Table 3 contained formatting errors. The corrected version is as below.

**Table 3:** Nonamer positions depicting amino acid differences between an HCSS nonamer and the corresponding variants, within and between the serotypes. Only positions of mutual information value of 1 and low entropy values are shown. The reference HCSS nonamers shown at the top were arbitrarily chosen (match to DENV2), and additional HCSS nonamers, if present, are shown in bold. Data for two additional positions are shown in Supplementary Table 4.

Protein | Entropy Value	NS1 | 0.12			NS2a | 0.11		
**HCSS Reference**	**146**	**NRAWNSLEV**	**154**	**202**	**LNPTAIFLT**	**210**
**DENV1**	146	Q....IW..	154	202	CK.LTM..I	210
	146	Q....VW..	154	202	CK.LPM..I	210
	146	L....IW..	154	202	CK.LTML.I	210
	146	H....IW..	154	202	CK.LTM.FI	210
	146	Q....IWK.	154	202	CK.LTMY.I	210
	146	Q..S.IW..	154	202	CK.L.M..I	210
	146	Q....IW.G	154	202	CK.L.ML.I	210
	146	Q....I...	154	202	CK.LTM..V	210
	146	Q...TIW..	154	202	CK.STM..I	210
				202	C..LTM..I	210
				202	SK.LTM..I	210
				202	CK.LTMYFI	210
				202	CKTLTM..I	210
**DENV2**	**146**	.........	**154**	**202**	.........	**210**
	146	S........	154	202	.......I.	210
	146	.......K.	154	202	.S.......	210
	146	.......KG	154	202	......L..	210
	146	..T.D....	154	202	......YF.	210
	146	.......KL	154			
**DENV3**	146	S....VW..	154	202	VP.LPL.IF	210
	146	A....VW..	154	202	VP.LPLLIF	210
	146	L....VW..	154	202	IP.LPL.IF	210
	146	S..L.VW..	154	202	.P.LPL.IF	210
				202	VQ.LPL.IF	210
				202	VS.LPL.IF	210
				202	VP.SPL.IF	210
				202	VPSLPL.IF	210
				202	AQ.LPL.IF	210
**DENV4**	146	R........	154	**202**	AQALPVY.M	**210**
	146	R....F...	154			
	146	R.....F..	154			
	146	R....FF..	154			
